# Is High-Density Lipoprotein Cholesterol Causally Related to Kidney Function? Evidence From Genetic Epidemiological Studies

**DOI:** 10.1161/ATVBAHA.116.308393

**Published:** 2016-10-26

**Authors:** Stefan Coassin, Salome Friedel, Anna Köttgen, Claudia Lamina, Florian Kronenberg

**Affiliations:** From the Division of Genetic Epidemiology, Department of Medical Genetics, Molecular and Clinical Pharmacology, Medical University of Innsbruck, Austria (S.C., S.F., C.L., F.K.); and Division of Genetic Epidemiology, Institute for Medical Biometry and Statistics, Medical Center – University of Freiburg, Faculty of Medicine, University of Freiburg, Germany (A.K.).

**Keywords:** atherosclerosis, dyslipidemia, genetic association study, HDL cholesterol, kidney, Mendelian randomization analysis

## Abstract

**Objective—:**

A recent observational study with almost 2 million men reported an association between low high-density lipoprotein (HDL) cholesterol and worse kidney function. The causality of this association would be strongly supported if genetic variants associated with HDL cholesterol were also associated with kidney function.

**Approach and Results—:**

We used 68 genetic variants (single-nucleotide polymorphisms [SNPs]) associated with HDL cholesterol in genome-wide association studies including >188 000 subjects and tested their association with estimated glomerular filtration rate (eGFR) using summary statistics from another genome-wide association studies meta-analysis of kidney function including ≤133 413 subjects. Fourteen of the 68 SNPs (21%) had a *P* value <0.05 compared with the 5% expected by chance (Binomial test *P*=5.8×10^−^^6^). After Bonferroni correction, 6 SNPs were still significantly associated with eGFR. The genetic variants with the strongest associations with HDL cholesterol concentrations were not the same as those with the strongest association with kidney function and vice versa. An evaluation of pleiotropy indicated that the effects of the HDL-associated SNPs on eGFR were not mediated by HDL cholesterol. In addition, we performed a Mendelian randomization analysis. This analysis revealed a positive but nonsignificant causal effect of HDL cholesterol–increasing variants on eGFR.

**Conclusions—:**

In summary, our findings indicate that HDL cholesterol does not causally influence eGFR and propose pleiotropic effects on eGFR for some HDL cholesterol–associated SNPs. This may cause the observed association by mechanisms other than the mere HDL cholesterol concentration.

## Abstract 2

Supplemental Digital Content is available in the text.

Chronic kidney disease (CKD) is characterized by oxidative stress, inflammation, and dyslipidemia.^1,2^ Besides various dyslipidemic changes, one can observe not only decreased high-density lipoprotein (HDL) cholesterol concentrations but also an impaired HDL maturation resulting in cholesterol ester–poor particles. These changes in quantitative amount and qualitative content of these multicomponent particles results not only in an impairment of its antioxidative and anti-inflammatory properties but also in a pronounced impairment of reverse cholesterol transport.^3^ The latter is responsible for relief of accumulated cholesterol in the blood vessel wall with transport of cholesterol to the liver for excretion or to organs in need of cholesterol. An impaired reverse cholesterol transport contributes not only to atherosclerosis but also to glomerulosclerosis and tubulointerstitial damage. Most evidence for these findings comes from experimental studies and was observed especially in more advanced stages of CKD.^2^

Epidemiological studies reported an association between low HDL cholesterol and poor kidney function, kidney function decline, or progression of CKD.^4–9^ Most of these studies included sample sizes of up to a few thousand individuals. Recently, Bowe et al^10^ investigated the association between HDL cholesterol concentrations and various CKD end points in a cohort of almost 2 million men from the United States Veterans with a median follow-up of 9 years. The authors observed an association between HDL cholesterol concentrations and these end points, with individuals showing low HDL cholesterol concentrations (<30 mg/dL) having the highest risk for CKD or CKD progression. However, epidemiological studies have the disadvantage that cause or consequence of an association of a certain biomarker with an outcome is difficult to disentangle. This is even true for prospective studies: it cannot be excluded that a biomarker is already influenced by the subclinical stage of the outcome long before the outcome becomes clinically evident. Therefore, the question remains whether a certain parameter is a risk marker or a risk factor for an outcome.

One approach is to perform interventional studies that influence the biomarker and to observe the influence on the outcomes. Unfortunately, this is time-consuming and would require the expensive development of a drug that influences the marker of interest. Mendelian randomization (MR) studies are a different and less-expensive option that could at least provide support for or against the development of interventional therapies. This study approach uses the fact that genetic variants having an influence on the marker of interest result in a lifelong exposure of lower or higher values of this biomarker.^11^ These genetic markers should therefore show an association with the outcome of interest if the association of the biomarker with the outcome is indeed causal.^12^

Mendelian randomization studies recently shed a new light on the strong association between HDL cholesterol concentrations and coronary heart disease. Previously, the stage had been set by many studies and finally by a large meta-analysis including >300 000 individuals free of vascular disease at baseline.^13^ This study found a 22% decrease in risk for coronary heart disease for each increase in HDL cholesterol of 15 mg/dL. If this association was causal, one would expect that patients free of coronary heart disease would carry more genetic variants that are associated with a lifelong exposure to higher HDL cholesterol values. However, this was not the case: after contrasting findings,^14^ recent large-scale studies dissecting the genetic architecture of lipid metabolism showed that increased low-density lipoprotein (LDL) cholesterol and triglyceride levels seem to be causally related to cardiovascular risk, but this was not the case for HDL cholesterol levels.^15–17^ This is in line with interventional trials with CETP (cholesteryl ester transfer protein) inhibitors that increased HDL cholesterol by >100% but did not decrease cardiovascular outcomes.^18^ It is now widely accepted consensus that HDL cholesterol rather represents a surrogate marker that does probably not properly reflect real HDL functionality. The HDL particle is indeed a highly complex entity carrying >80 different proteins, some hundred lipid species^19^ and a dozen micro-RNAs,^20^ and recent studies^19,21^ revealed the existence of dysfunctional HDL particles,^22^ which lose their atheroprotective effect because of pathological modifications of their composition.^21^ This indicates that the traditional good HDL, bad LDL hypothesis is indeed too simplistic.

Considering the long-lasting and at the final end disappointing developments on HDL cholesterol concentrations and cardiovascular events raises concerns to experience a déjà vu with HDL cholesterol and kidney function or CKD progression. One possibility to shorten the way is to use publically available data from genome-wide association studies (GWAS) on HDL cholesterol and kidney function.

Starting from the findings of Bowe et al, we aimed to investigate whether HDL cholesterol is causally related to kidney function. The causality of an association between HDL cholesterol and kidney function would indeed be strongly supported if genetic variants that are associated with higher HDL cholesterol concentrations were also associated with lower kidney function. Therefore, we used single-nucleotide polymorphisms (SNPs) that were found to be associated with HDL cholesterol in publically available GWAS and tested whether they were also associated with kidney function in summary statistics from another publically available GWAS. On the basis of this summarized data, we performed a Mendelian randomization analysis.

## Materials and Methods

Materials and Methods are available in the online-only Data Supplement. In brief, we selected genetic variants that were found to be associated with HDL cholesterol on a genome-wide significant level (*P*<5×10^−^^8^) published by the Global Lipids Genetics Consortium including >188 000 subjects.^23^ We decided not to include SNPs that were found to be genome-wide significant exclusively with other lipid phenotypes than HDL cholesterol. With this approach, weak instrument bias can be held as small as possible. These SNPs were looked up in publicly available GWAS summary statistics from the CKDGen Consortium for the association with kidney function parameters including ≤133 413 subjects.^24^ Over-representation of *P* values <0.05 was tested using a binomial test. The significance level of the single SNP look-up was set to 0.05/68=7.35×10^−^^4^ after Bonferroni correction on the number of SNPs. In addition, a Mendelian randomization analysis was performed using the published summarized data as described in the study by Burgess et al.^25^ A schematic overview of the study design, main findings, and interpretation is provided in Figure I in the online-only Data Supplement.

## Results

### Single SNP Associations

**Figure 1.**
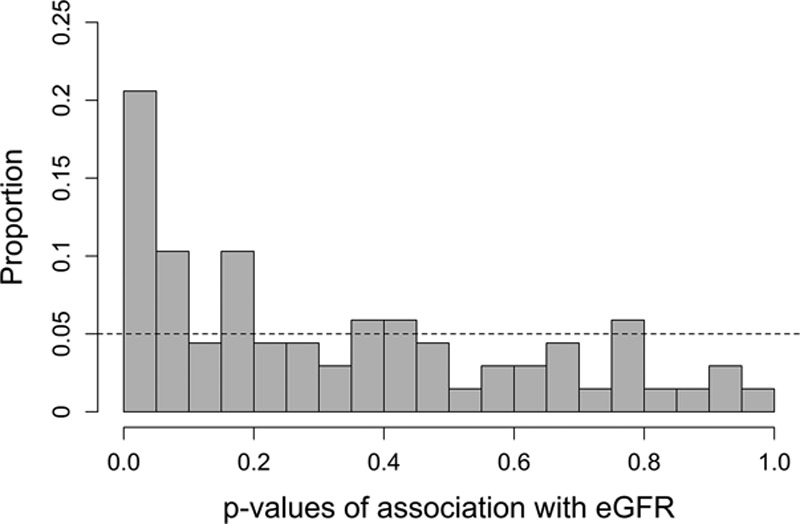
Distribution of *P* values for estimated glomerular filtration rate (eGFR) using all 68 SNPs; under the null hypothesis of no association, a uniform distribution would have been expected, indicated by the dashed line.

**Figure 2.**
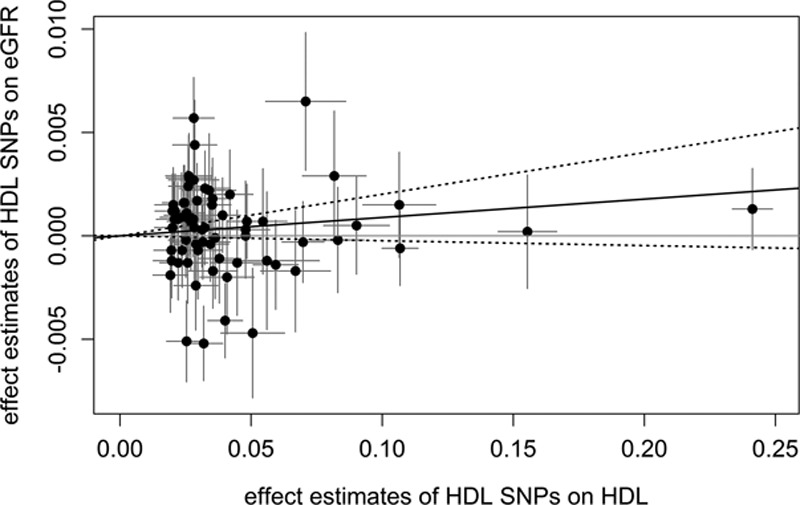
Scatter plots showing the effect estimates of single-nucleotide polymorphism (SNP)–high-density lipoprotein (HDL) associations (±95% confidence interval [CI]) in SD of HDL cholesterol values on the x axis and SNP–estimated glomerular filtration rate (eGFR) associations (±95% CI) in log-transformed eGFR values on the y axis for all 68 SNPs. The continuous black line represents the Mendelian randomization estimate of HDL on eGFR, the dashed lines the corresponding 95% CI.

**Figure 3.**
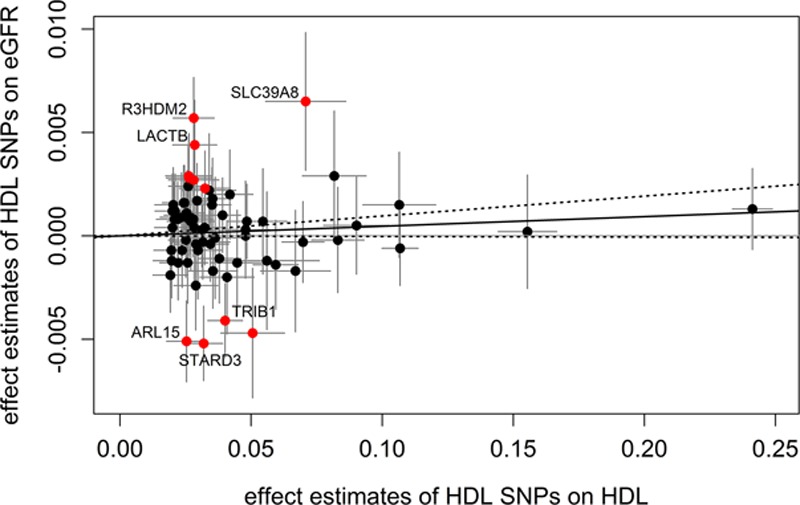
Scatter plots showing the effect estimates of single-nucleotide polymorphism (SNP)–high-density lipoprotein (HDL) associations (±95% confidence interval [CI]) in SD of HDL cholesterol values on the x axis and SNP–estimated glomerular filtration rate (eGFR) associations (±95% CI) in log-transformed eGFR values on the y axis. SNPs showing effects that are potentially not mediated by HDL cholesterol (according to goodness-of-fit test) are marked in red. For the Mendelian randomization estimate, these SNPs were excluded. The continuous black line represents the Mendelian randomization estimate of HDL on eGFR, the dashed lines the corresponding 95% CI. The 6 SNPs, which were significantly associated in a single SNP analysis with eGFR, are annotated with their gene name.

Results for all 68 individual and independent SNPs that were reported to be associated with HDL cholesterol concentrations^23^ are shown in Table I in the online-only Data Supplement. For each associated locus, the lead SNP was selected in the first place irrespective of possible associations with other lipid phenotypes. Under the assumption that there is no association of HDL cholesterol–associated SNPs with estimated glomerular filtration rate (eGFR), a uniform distribution of *P* values would be expected. Figure 1 shows the observed distribution of the *P* values for the association analysis between the HDL cholesterol–associated SNPs and eGFR. There was a considerable excess of low *P* values: 14 of 68 SNPs (21%) had a *P* value <0.05, compared with the 5%, which would have been expected (Binomial test *P*=5.8×10^−^^6^). Of these, 6 SNPs still passed a significance level corrected for the number of evaluated SNPs (Bonferroni correction, 0.05/68=7.35×10^−^^4^; Table 1). Two of these even reached genome-wide significance for eGFR (rs11869286 and rs11613352). Interestingly, the genetic variants with the strongest associations with HDL cholesterol concentrations were not the same as those with the strongest association with kidney function and vice versa. A side-by-side comparison of the SNP effect estimates on HDL cholesterol and eGFR can be found in Table I in the online-only Data Supplement and is shown in Figures 2 and 3. The effects on HDL cholesterol of the 6 SNPs, which we detected to be associated with both HDL cholesterol and eGFR, were rather modest. They were the 8th, 21st, 32nd, 39th, 41st, and 53rd SNP among the HDL cholesterol–associated SNPs in Global Lipids Genetics Consortium on the β-estimate on HDL cholesterol. Conversely, *CETP*, which showed the largest β on HDL cholesterol in the Global Lipids Genetics Consortium (rs3764261; β= 0.241; *P*=1×10^−^^769^), was not significantly associated with eGFR. Notably, 6 SNPs (mapping in or near the genes *TRIB1*, *STARD3*, and *ARL15*) were not consistent with the direction of effects, which would have been expected from the results of Bowe et al^10^ in the sense that SNPs lowering HDL cholesterol were not associated with lower eGFR.

**Table 1.**
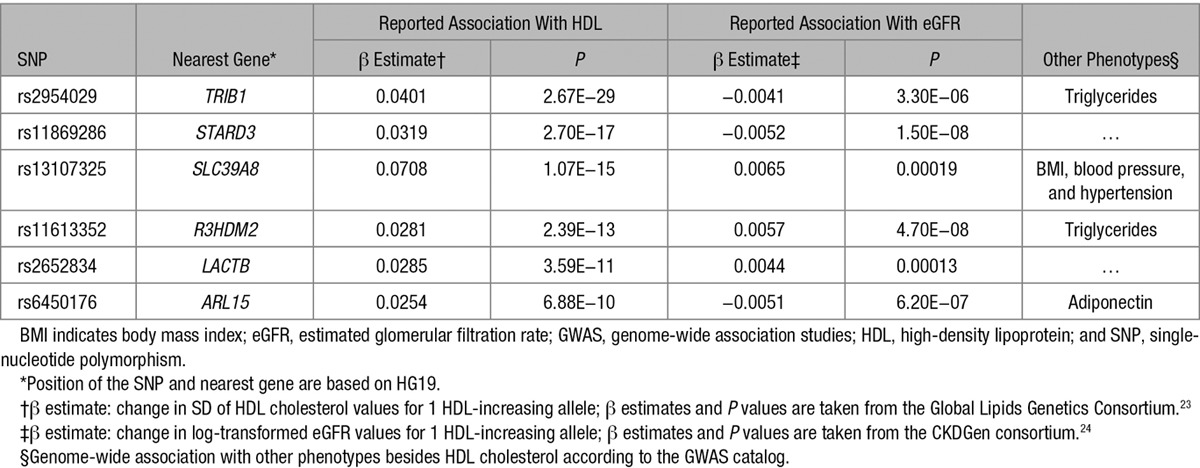
Summary of the Single SNP Association Results From Published Data for All 6 SNPs, Which Were Significantly Associated in a Single SNP Analysis With eGFR

Twenty-eight of the 68 HDL cholesterol–associated SNPs (41%) were found to have genome-wide significant associations with other phenotypes besides HDL cholesterol according to the GWAS catalog (Table I in the online-only Data Supplement), primarily with triglyceride levels indicating potential pleiotropy.

### Mendelian Randomization Analysis

To estimate the effect of genetically increased HDL cholesterol values on eGFR, single SNP effects were combined using a meta-analysis approach. Because only summarized data are present, the *F* statistic and the coefficient of determination (*R*^2^)—which are measures of the strength of the instrumental variables (IV)—cannot be determined empirically using this data. However, only genome-wide significant SNPs that are independent of each other (pairwise linkage disequilibrium between all SNPs: *r*^2^<0.1) were included in this analysis (*P*<5×10^−8^). This corresponds to an *F* statistic >30 for each variant.^25^ In the Mendelian randomization literature, a threshold of *F*<10 has typically been used to define a weak IV (the Staiger–Stock rule.^26,27^) It has been reported that SNPs identified in GWAS on HDL cholesterol roughly explain 13% of the phenotypic variance.^23,28^ Calculating the proportion of explained variance for the 68 used SNPs in our analysis based on the reported β estimates yielded an estimate of 6.6%. Even for this lower conservative estimate, weak instrument bias can be assumed to be low.^25^ Using the adapted MR-Egger regression, no directional bias could be detected (MR-Egger intercept=0.0003; 95% confidence interval [CI]=−0.0005 to 0.0010; *P*=0.4872; Table 2). Nevertheless, when including all 68 SNPs, high heterogeneity was detected, necessitating the use of a random effects model.

**Table 2.**
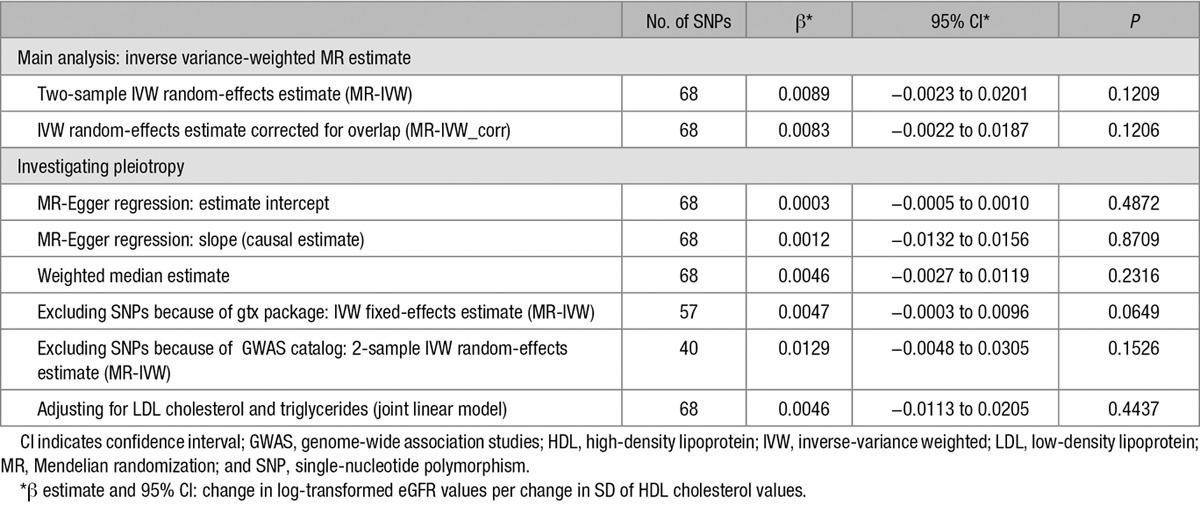
Overview of Causal Estimates Obtained From the Different Approaches in MR

Figure 2 shows all single SNP effect estimates on both the HDL cholesterol concentrations and eGFR. The straight black line displays the combined Mendelian randomization estimate. There was a small positive effect, which means that eGFR was higher for genetically increased HDL cholesterol values. However, this effect was not significant (2-sample β_Mendelian randomization−inverse-variance weighted [MR-IVW]_=0.0089; 95% CI=−0.0023 to 0.0201; *P*=0.1209; *P*_heterogeneity_<10^−^^16^) even when we corrected for overlapping samples in the GWAS of HDL cholesterol and eGFR (β_MR-IVW_corr_=0.0083 95% CI=−0.0022 to 0.0187; *P*=0.1206; *P*_heterogeneity_<10^−^^16^). Also, the causal estimate obtained from the MR-Egger regression showed no effect (β_MR-Egger_=0.0012; 95% CI=−0.0132 to 0.0156; *P*=0.8709; Figure II in the online-only Data Supplement). Likewise, the weighted median estimate approach resulted in a nonsignificant causal estimate of β_WME_=0.0046(95% CI=−0.0027 to 0.0119; *P*=0.2316). Taken together, these results argue against a causal effect of HDL cholesterol on eGFR (Table 2).

To further examine the heterogeneity and possible pleiotropic effects, we conducted a goodness-of-fit test. This resulted in 11 SNPs that could be detected to influence eGFR through additional pathways not involving HDL cholesterol concentrations. These SNPs included all 6 SNPs, which were significantly associated with eGFR in the single SNP analysis (*TRIB1*, *STARD3*, *SLC39A8*, *R3HDM2*, *LACTB*, and *ARL15*). Removing these 11 SNPs also removed heterogeneity. The subsequently performed fixed-effects inverse variance model yielded a positive, but nonsignificant effect (β_MR-IVW_=0.0047; 95% CI=−0.0003 to 0.0096; *P*=0.0649; *P*_heterogeneity_=0.0556; Figure 3). Removing potential pleiotropic effects according to a search in the GWAS catalog led to the exclusion of 28 SNPs and to a similar effect estimate (2-sample β_MR-IVW_=0.0129; 95% CI=−0.0048 to 0.0305; *P*=0.1526; *P*_heterogeneity_=3.75×10^−^^9^; Figure III in the online-only Data Supplement). Finally, adjusting for the effect estimates of HDL cholesterol SNPs on low-density lipoprotein cholesterol and triglycerides resulted in an estimate of the causal effect of β_jointlinear model_=0.0046 (95% CI=−0.0113 to 0.0205; *P*=0.4437). An overview of all estimates calculated in the different sets of SNPs is given in Table 2.

## Discussion

In this study, we followed up a recently published association of HDL cholesterol levels and eGFR by investigating whether genetic variants that show genome-wide significant associations with HDL cholesterol concentrations are also associated with kidney function. For this purpose, we (1) assessed whether HDL cholesterol–associated SNPs are enriched for SNPs associated with kidney function and (2) used a Mendelian randomization analysis to assess whether the observational association between higher HDL cholesterol and better kidney function may be causal.

We observed a higher fraction of HDL cholesterol–associated variants to be also associated with eGFR than expected by chance. After Bonferroni correction, 6 SNPs were significantly associated with eGFR. Two of them were genome-wide significant, mapping to 2 previously found regions for kidney function.^24,29^ Interestingly, the genetic variants with the strongest associations with HDL cholesterol concentrations were not the same as those with the strongest association with kidney function and vice versa.

Second, the Mendelian randomization resulted in a positive but not significant causal effect. This indicates that eGFR is not significantly higher in people with genetically increased HDL cholesterol concentrations. Using the MR-Egger regression, no directional pleiotropy could be detected. Nevertheless, unbalanced pleiotropy cannot be ruled out using this method. The more consistent causal estimate in case of pleiotropy obtained from the slope of the MR-Egger regression strengthened the finding that HDL cholesterol does not causally influence eGFR.

**Figure 4.**
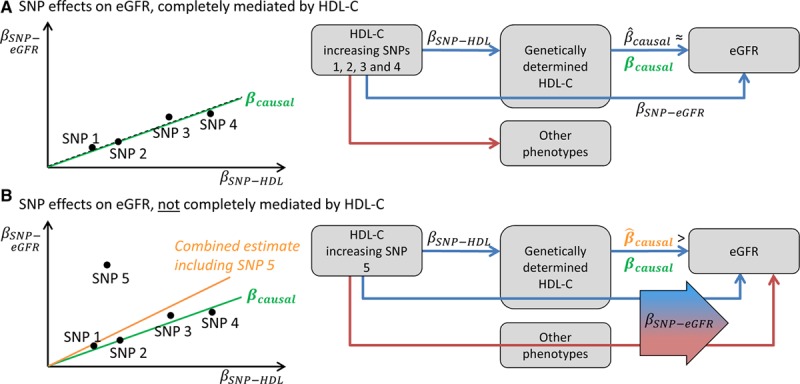
Schematic illustration of the mechanism by which one pleiotropic single-nucleotide polymorphism (SNP) can introduce bias in the Mendelian randomization analysis: **A**, Let us assume 4 SNPs, which increase high-density lipoprotein cholesterol (HDL-C). They can also be associated with other phenotypes, but when the other phenotypes are not associated with estimated glomerular filtration rate (eGFR), the estimated causal effect estimates for each SNP 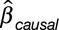 approximates the true unknown causal effect *β_causal_*. In a scatter plot showing the effects of the SNPs on HDL-C ( *β_SNP-HDL_*) together with their effects on eGFR ( *β_SNP-eGFR_*), these SNPs approximately lie on a line, which slope represents the causal effect. **B**, One SNP is added to this example with pleiotropic effects on another phenotype, which is also associated with eGFR. Then, the effect of the SNP on eGFR ( *β_SNP-eGFR_*) is not mediated completely by HDL-C but also includes the effect of the other phenotype on eGFR. In this case, 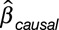 is higher than *β_causal_* for this SNP, and heterogeneity will be introduced in the meta-analysis of the Mendelian randomization estimates. In this example, if this SNP is not excluded, the combined estimate will overestimate the true causal estimate.

Removing potentially pleiotropic SNPs identified by the GWAS catalog increased variability of the estimated effect resulting in a wider confidence interval of the causal effect, because most of the SNPs with high effects on HDL cholesterol are also associated with other phenotypes (primarily with triglyceride levels). However, as explained in Figure 4, pleiotropic effects only represent a problem for a Mendelian randomization analysis if a SNP impacts eGFR also independently from HDL cholesterol, or in other words, in case that the effect on eGFR is not entirely mediated by HDL cholesterol. Removing the SNPs identified to be pleiotropic by the goodness-of-fit test attenuated the effect estimate but also reduced variability, yielding a positive but still not statistically significant effect. Even if the effect was significant, a causal effect of such small magnitude would not explain the observed epidemiological association of HDL cholesterol on kidney function.^10^ The effect estimate derived from this method is similar in magnitude with the estimates obtained from weighted median method and for the regression adjusting for low-density lipoprotein cholesterol and triglycerides. Therefore, all performed methods that allow and/or adjust for potential pleiotropy come to the conclusion that HDL cholesterol does not causally influence eGFR.

In light of the Mendelian randomization data, the observed over-representation of eGFR-associated SNPs among the HDL-associated SNPs, which we observed in our first analysis, has to be critically evaluated. Besides an impact of confounding through triglyceride concentrations, the associations might reflect regulators of HDL function and maturation rather than mere HDL cholesterol concentration. Keeping that in mind, it might not be contradicting that 3 of the significant SNPs in *ARL15*, *TRIB1*, and *STARD3* do not match the expected effect direction, which is eGFR increasing for HDL cholesterol–increasing alleles. The observed association with eGFR might not follow the same pathway as the association with HDL cholesterol, indicating the possibility of pleiotropic effects. Unfortunately, little is known about the function of the significant genes in HDL and kidney metabolism (Note I in the online-only Data Supplement).

One limitation of our study is that no individual-level data but only summarized data based on published GWAS studies were available for analysis. Therefore, the strength of the used instruments and the power of the Mendelian randomization analysis cannot be measured directly. Hence, a possible bias caused by weak instruments cannot be estimated. Assuming a 2-sample setting, using weak instruments would lead to an estimate of the causal effect that is biased toward the null hypothesis of no effect.^30,31^ In a one-sample setting, weak instruments would lead to an estimate of the causal effect that is biased toward the observational effect estimate. Because we have ≈43% overlap between both samples, we would assume a possible bias between a null and observed effect. We, therefore, included a correction for the sample overlap. Furthermore, because all SNPs are genome-wide significantly associated with HDL cholesterol, the probability for weak instruments is low. Using summarized data does not only have disadvantages. As shown by Burgess et al,^25^ Mendelian randomization estimates derived from summarized data are similarly efficient as estimates derived from individual-level data. Using summarized data including huge sample sizes increases power in a way that would not be possible by using individual-level data only, given restrictions with sharing individual-level data. Additionally, using large sample sizes increases precision of the estimates.

Was our study limited by power? If we assume that there is a causal relationship between HDL cholesterol and eGFR and that the real causal effect is higher than one third of the observed effect, one would expect that we would have found it (for details, see Note II in the online-only Data Supplement).

Another limitation of our analysis is that our applied Mendelian randomization approach assumes a linear relationship of the intermediate phenotype (HDL cholesterol) on the outcome (eGFR). Bowe et al^10^ showed such a linear relationship for the cross-sectional part of their study. However, the analysis on incident CKD and disease progression, adjusted for baseline eGFR values, showed a U-form, but still with the highest risk for low HDL cholesterol values. If there was a nonlinear causal effect of HDL cholesterol on eGFR, we would not have found it with the Mendelian randomization approach based on published summarized data. The published GWAS results for eGFR are based on cross-sectional studies, which are primarily population based. Therefore, we are not able to evaluate a causal effect of the SNPs on kidney disease progression.

In conclusion, our findings do not confirm a causal effect of HDL cholesterol on kidney function, although we observed a significant over-representation of low eGFR-associated *P* values for HDL cholesterol SNPs. If there was indeed an association of these individual HDL cholesterol–associated SNPs with eGFR, it might be explained by other culprits than the cholesterol content of the HDL particle.

## Sources of Funding

This work was supported by the Austrian Science Fund (FWF) (Project P 26660-B13) to C. Lamina. The work of A. Köttgen was supported through a Heisenberg Professorship of the German Research Foundation (KO 3598/3-1).

## Disclosures

None.

## Supplementary Material

## Highlights

- Publically available data from large genome-wide association studies were used to test whether genetic variants influencing high-density lipoprotein cholesterol levels have an influence on kidney function.
- We observed a significantly higher frequency of high-density lipoprotein cholesterol single-nucleotide polymorphisms to be associated with kidney function (21% versus 5%).
- The genetic variants with the strongest associations with high-density lipoprotein cholesterol concentrations were not the same as those with the strongest association with kidney function.
- A Mendelian randomization analysis did not confirm a significant causal effect of high-density lipoprotein cholesterol–increasing variants on estimated glomerular filtration rate.
