# Supplementary material for: Is High-Density Lipoprotein Cholesterol Causally Related to Kidney Function? Evidence From Genetic Epidemiological Studies
Source: Arterioscler Thromb Vasc Biol. 2016 Oct 26;36(11):2252–8. doi: 10.1161/ATVBAHA.116.308393 (PMC5084637; doi:10.1161/ATVBAHA.116.308393)
Supplement: Supplementary file 1 [file atv-36-2252-s001.pdf]

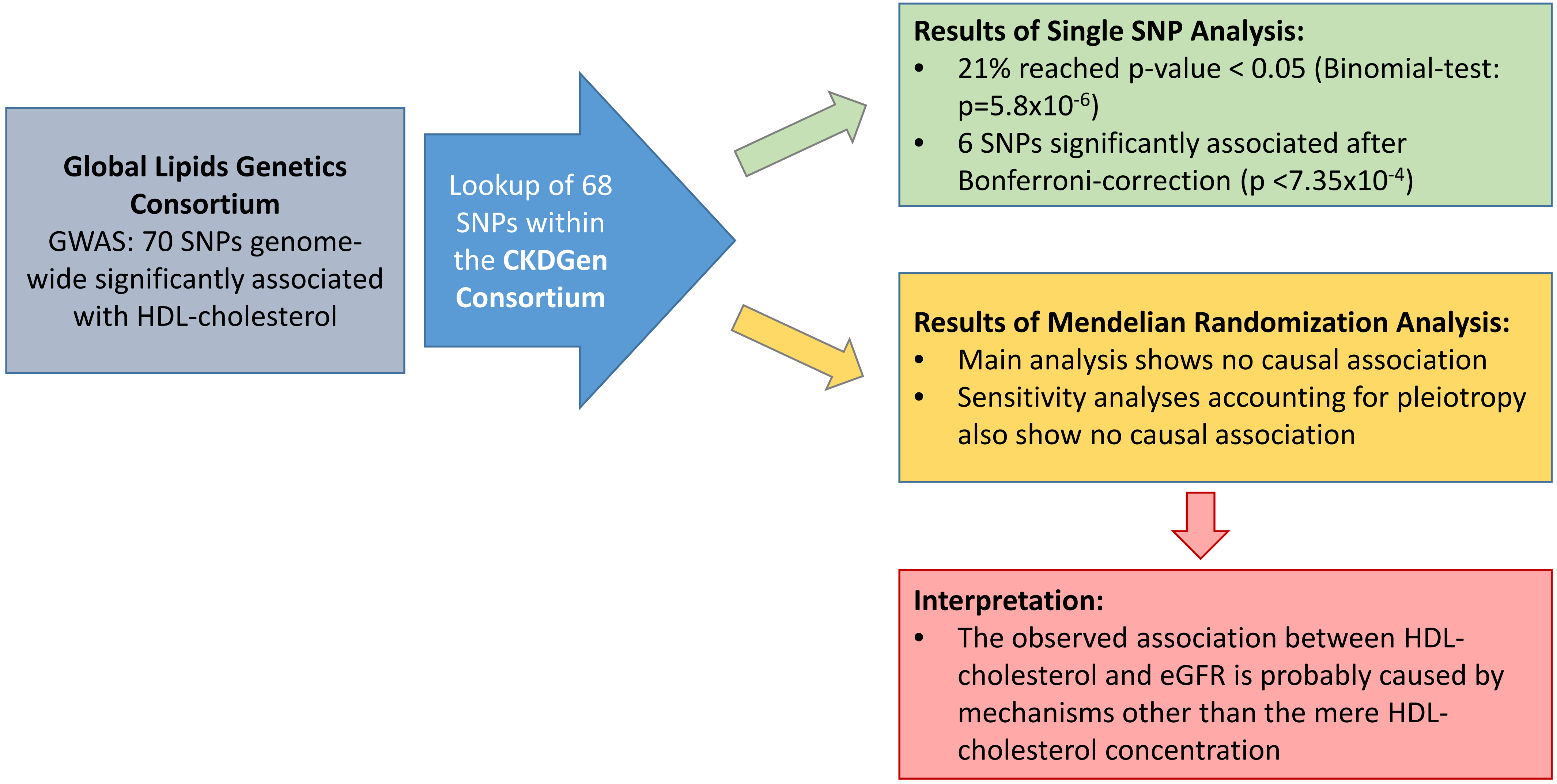
Global Lipids Genetics Consortium
GWAS: 70 SNPs genome-wide significantly associated with HDL-cholesterol
Lookup of 68 SNPs within the CKDGen Consortium
Results of Single SNP Analysis:
• 21% reached p-value < 0.05 (Binomial-test: p=5.8x10-6)
• 6 SNPs significantly associated after Bonferroni-correction (p <7.35x10-4)
Results of Mendelian Randomization Analysis:
• Main analysis shows no causal association
• Sensitivity analyses accounting for pleiotropy also show no causal association
Interpretation:
• The observed association between HDL-cholesterol and eGFR is probably caused by mechanisms other than the mere HDL-cholesterol concentration
