# Supplementary material for: Is High-Density Lipoprotein Cholesterol Causally Related to Kidney Function? Evidence From Genetic Epidemiological Studies
Source: Arterioscler Thromb Vasc Biol. 2016 Oct 26;36(11):2252–8. doi: 10.1161/ATVBAHA.116.308393 (PMC5084637; doi:10.1161/ATVBAHA.116.308393)
Supplement: Supplementary file 2 [file atv-36-2252-s002.pdf]

Supplement Material to

Is HDL-cholesterol causally related to kidney function? Evidence from genetic epidemiological studies

Running title: HDL-cholesterol genes and kidney function

Stefan Coassin* [1], Salome Friedel* [1], Anna Köttgen [2],
Claudia Lamina [1], Florian Kronenberg [1]

* These authors contributed equally

[1] Division of Genetic Epidemiology, Department of Medical Genetics, Molecular and Clinical Pharmacology, Medical University of Innsbruck, Innsbruck, Austria

[2] Division of Genetic Epidemiology, Institute for Medical Biometry and Statistics, Medical Center – University of Freiburg, Faculty of Medicine, University of Freiburg, Germany

Supplementary Note I

Only *TRIB1* [1-3] has been studied with respect to kidney function. *TRIB1* expression has been shown to predict serum creatinine at follow-up in patients with proteinuric renal disease [4] and might also represent a biomarker for allograft rejection in kidney transplantation [5]. An interaction between ApoL1 effect and variants near *TRIB1* has been reported in APOL1-associated nephropathy [6]. The observation that both HDL-cholesterol efflux capacity [7] and *TRIB1* expression [5] are associated with allograft rejection and failure might indirectly link one of our top hits to HDL function, which is known to be also strongly modified in kidney disease [8]. *STARD3* overexpression increases lipidation of exogenous apoA-I [9], but effects on kidney metabolism have not been reported. However, FBXL20, located about 400 kb upstream, has been implicated with kidney function by CKDGen Consortium GWAS [10, 11] and the regional plots in [10] show a modest correlation between STARD3 and FBXL20. Conversely, *R3HDM2* and its neighboring gene INHBC have been associated with serum urate levels in previous studies [12, 13]. INHBC has been previously reported by the CKDGen Consortium [10]. Our SNP maps in the intergenic region between these two genes. No function or phenotype is known for *ARL15*, but it has been proposed to be structurally related to ADP-ribosylation factors and Ras-related GTP-binding proteins which play key roles in the regulation of intracellular vesicle trafficking [14]. Also STARD3 has been recently linked to late-endosome trafficking [15].

Generally, the assignment of a SNP association to any gene has to be treated with care. This applies especially for SNPs in intergenic regions without clear hints about the gene whose function is modified by the SNP.

Supplementary Note II: Power calculations

Power calculations were performed using the online tool https://sb452.shinyapps.io/power/. The tool requires an estimate of the proportion of HDL variance explained by the SNPs, as well as the expected causal estimate, which one wants to detect with the given sample size based on change in one standard deviation (SD) of the outcome (eGFR) per one SD change in exposure (HDL-cholesterol). The summarized data obtained from the GLGC consortium provide beta estimates per change in SD of HDL-cholesterol, but the beta estimates from the CKDGen Consortium refer to change in one unit of log-transformed eGFR. Therefore, the causal effect estimates calculated with the Mendelian randomization approach using summarized data from both consortia is not usable for power calculations, and we instead used an observed effect of ~0.08 SD change in eGFR per 1 SD change of HDL-cholesterol based on data from population-based studies. Assuming a combined R^2 of 6.6% for all 68 SNPs (calculated based on reported beta estimates as described in the Methods section) and 6.2% for 57 SNPs (excluding pleiotropic SNPs), a sample size of ~133000 and a

significance level of 5%, we have 100% power to detect a causal effect of this size, if it is truly there. We have 80% power to detect a causal effect of 0.03, which is roughly one third of the observed effect. Of note, our calculated causal effects range between 0.0012 to 0.013 (depending on the methods) and cannot be compared to the effect assumed for the power analysis, since it is based on different units.

Supplementary Table I: Summary of the single SNP association results from published data for all SNPs

SNP	Nearest Gene[+]	chr	Position[+]	A1*	A2	EAF[$]	Association results with HDL based on Willer et al. [16] β[#]	se	p	Association results with eGFR based on Pattaro et al. [11] β[##]	se	p
rs3764261	CETP	16	56993324	a	c	0.2942	0.2412	0.0039	1.00E-769	0.0013	0.0010	0.2
rs1532085	LIPC	15	58683366	a	g	0.3668	0.1068	0.0035	1.24E-188	-0.0006	0.0009	0.5
rs12678919	LPL	8	19844222	g	a	0.1214	0.1554	0.0057	1.38E-149	0.0002	0.0014	0.91
rs1883025	ABCA1	9	107664301	c	t	0.7573	0.0698	0.0041	1.50E-65	-0.0003	0.0010	0.8
rs16942887	PSKH1	16	67928042	a	g	0.1332	0.0831	0.0051	8.28E-54	-0.0002	0.0013	0.87
rs964184	ZNF259	11	116648917	c	g	0.8380	0.1065	0.0071	6.09E-48	0.0015	0.0013	0.25
rs7241918	LIPG	18	47160953	t	g	0.8232	0.0902	0.0064	1.11E-44	0.0005	0.0012	0.69
rs9987289	PPP1R3B	8	9183358	g	a	0.9248	0.0817	0.0062	1.95E-41	0.0029	0.0016	0.069
rs4846914	GALNT2	1	230295691	a	g	0.5844	0.0479	0.0034	3.51E-41	0.0000	0.0009	0.99
rs6065906	PCIF1	20	44554015	t	c	0.8021	0.0594	0.0044	5.34E-40	-0.0014	0.0011	0.22
rs838880	SCARB1	12	125261593	c	t	0.3259	0.0484	0.0039	6.38E-32	0.0007	0.0009	0.5
rs2954029	TRIB1	8	126490972	t	a	0.4683	0.0401	0.0034	2.67E-29	-0.0041	0.0009	3.30E-06
rs3136441	F2	11	46743247	c	t	0.1372	0.0545	0.0047	6.76E-29	0.0007	0.0013	0.6
rs174546	FADS1	11	61569830	c	t	0.6425	0.0391	0.0035	8.30E-28	0.0010	0.0009	0.28
rs386000	LILRA3	19	54792761	c	g	0.1992	0.0479	0.0047	2.71E-23	0.0003	0.0012	0.78
rs4420638	APOC1	19	45422946	a	g	0.8140	0.0669	0.0068	1.72E-21	-0.0017	0.0015	0.27
rs1689800	ZNF648	1	182168885	a	g	0.6728	0.0344	0.0036	4.77E-20	-0.0004	0.0009	0.63
rs581080	TTC39B	9	15305378	c	g	0.8206	0.0419	0.0045	1.04E-19	0.0020	0.0011	0.082
rs2925979	GAN	16	81534790	c	t	0.7045	0.0351	0.0037	1.32E-19	0.0015	0.0009	0.12
rs4660293	PABPC4	1	40028180	a	g	0.7639	0.0353	0.0040	2.86E-18	0.0018	0.0010	0.084
rs181362	RIMBP3C	22	21932068	c	t	0.8008	0.0379	0.0042	4.30E-18	-0.0011	0.0011	0.3
rs2972146	IRS1	2	227100698	g	t	0.3773	0.0323	0.0035	1.85E-17	0.0004	0.0009	0.67
rs11869286	STARD3	17	37813856	c	g	0.6755	0.0319	0.0037	2.70E-17	-0.0052	0.0009	**1.50E-08**
rs2293889	TRPS1	8	116599199	g	t	0.5871	0.0312	0.0035	4.27E-17	0.0003	0.0009	0.72
rs4731702	KLF14	7	130433384	t	c	0.4604	0.0294	0.0034	4.84E-17	0.0017	0.0009	0.06
rs12748152	PIGV	1	27138393	c	t	0.9288	0.0506	0.0062	9.74E-16	-0.0047	0.0016	0.0044
rs13107325	SLC39A8	4	103188709	c	t	0.9222	0.0708	0.0078	1.07E-15	0.0065	0.0017	0.00019
rs12328675	COBLL1	2	165540800	c	t	0.1491	0.0447	0.0052	2.13E-15	-0.0013	0.0013	0.32
rs7941030	UBASH3B	11	122522375	c	t	0.3971	0.0269	0.0035	1.12E-14	0.0009	0.0009	0.33
rs7134594	MMAB	12	110000193	t	c	0.5554	0.0354	0.0048	1.77E-13	-0.0017	0.0009	0.059
rs11613352	R3HDM2	12	57792580	t	c	0.1913	0.0281	0.0040	2.39E-13	0.0057	0.0010	**4.70E-08**
rs17695224	FPR3	19	52324216	g	a	0.7612	0.0290	0.0039	2.42E-13	-0.0004	0.0010	0.66
rs17145738	TBL2	7	72982874	t	c	0.1174	0.0408	0.0053	4.95E-13	-0.0020	0.0014	0.16
rs4148008	ABCA8	17	66875294	c	g	0.6913	0.0280	0.0038	1.13E-12	0.0008	0.0009	0.42
rs3822072	FAM13A	4	89741269	g	a	0.5119	0.0251	0.0034	4.06E-12	-0.0002	0.0009	0.82
rs702485	DAGLB	7	6449272	g	a	0.4499	0.0243	0.0034	6.45E-12	0.0016	0.0009	0.076

… to be continued

Supplementary Table I: continued

SNP	Nearest Gene[+]	chr	Position[+]	A1*	A2	EAF[$]	Association results with HDL based on Willer et al. [16] β[#]	se	p	Association results with eGFR based on Pattaro et al. [11] β[##]	se	p
rs2013208	SEMA3F	3	50129399	t	c	0.5053	0.0254	0.0036	8.92E-12	0.0011	0.0009	0.23
rs4142995	SNX13	7	17919258	g	t	0.6161	0.0263	0.0037	9.37E-12	0.0008	0.0009	0.37
rs4129767	PGS1	17	76403984	a	g	0.5237	0.0237	0.0034	2.10E-11	-0.0007	0.0009	0.43
rs998584	VEGFA	6	43757896	c	a	0.4855	0.0260	0.0038	2.27E-11	0.0024	0.0010	0.019
rs2652834	LACTB	15	63396867	g	a	0.7652	0.0285	0.0043	3.59E-11	0.0044	0.0011	0.00013
rs13326165	STAB1	3	52532118	a	g	0.1873	0.0289	0.0043	9.04E-11	-0.0024	0.0011	0.034
rs11246602	OR4C46	11	51512090	c	t	0.1332	0.0340	0.0052	1.68E-10	0.0022	0.0014	0.12
rs970548	MARCH8	10	46013277	c	a	0.2770	0.0258	0.0039	1.71E-10	-0.0013	0.0010	0.19
rs1936800	RSPO3	6	127436064	c	t	0.5277	0.0200	0.0034	3.06E-10	0.0012	0.0009	0.17
rs6450176	ARL15	5	53298025	g	a	0.7216	0.0254	0.0039	6.88E-10	-0.0051	0.0010	6.20E-07
rs4765127	ZNF664	12	124460167	t	g	0.3628	0.0324	0.0050	7.79E-10	0.0023	0.0009	0.011
rs731839	PEPD	19	33899065	a	g	0.6583	0.0220	0.0037	3.44E-09	0.0008	0.0009	0.37
rs2290547	SETD2	3	47061183	g	a	0.7889	0.0297	0.0046	3.69E-09	-0.0007	0.0012	0.56
rs13076253	CPNE4	3	131751775	a	c	0.8522	0.0283	0.0048	4.96E-09	0.0004	0.0013	0.78
rs4650994	C1orf220	1	178515312	g	a	0.5172	0.0210	0.0034	6.70E-09	0.0012	0.0009	0.16
rs1121980	FTO	16	53809247	g	a	0.5528	0.0196	0.0034	6.79E-09	-0.0007	0.0009	0.45
rs4983559	ZBTB42	14	105277209	g	a	0.3773	0.0197	0.0036	9.57E-09	-0.0012	0.0009	0.19
rs4917014	IKZF1	7	50305863	g	t	0.3404	0.0222	0.0036	1.03E-08	-0.0013	0.0009	0.16
rs7134375	PDE3A	12	20473758	a	c	0.4169	0.0207	0.0035	1.05E-08	0.0008	0.0009	0.39
rs499974	MOGAT2	11	75455021	c	a	0.8245	0.0263	0.0044	1.12E-08	0.0028	0.0011	0.016
rs6805251	GSK3B	3	119560606	t	c	0.3813	0.0200	0.0035	1.33E-08	0.0004	0.0009	0.64
rs12145743	C1orf66	1	156700651	g	t	0.3311	0.0203	0.0036	1.80E-08	0.0015	0.0009	0.11
rs7255436	ANGPTL4	19	8433196	a	c	0.5435	0.0316	0.0053	1.87E-08	-0.0003	0.0009	0.76
rs17173637	ABP1	7	150529449	t	c	0.9024	0.0363	0.0057	1.90E-08	-0.0001	0.0015	0.95
rs605066	CITED2	6	139829666	t	c	0.5620	0.0281	0.0049	2.79E-08	0.0027	0.0009	0.0027
rs4759375	SBNO1	12	123796238	t	c	0.0937	0.0560	0.0102	3.01E-08	-0.0012	0.0017	0.49
rs12801636	PCNXL3	11	65391317	a	g	0.2243	0.0235	0.0042	3.15E-08	0.0009	0.0011	0.41
rs12967135	MC4R	18	57849023	g	a	0.7691	0.0262	0.0045	3.57E-08	0.0029	0.0010	0.0056
rs2606736	ATG7	3	11400249	c	t	0.3945	0.0246	0.0043	4.80E-08	0.0016	0.0009	0.09
rs10019888	RBPJ	4	26062990	a	g	0.8364	0.0270	0.0046	4.90E-08	0.0007	0.0012	0.55
rs2602836	ADH5	4	100014805	a	g	0.4274	0.0192	0.0034	4.96E-08	-0.0019	0.0009	0.032
rs2923084	ADM	11	10388782	a	g	0.8470	0.0256	0.0045	5.00E-08	0.0010	0.0011	0.37

[+]Position of the SNP and nearest gene are based on HG19; *A1:effect allele (HDL-increasing); [#] β estimate: change in s.d. of inverse normally transformed HDL cholesterol values for one HDL-increasing allele; [##] β estimate: change in log-transformed eGFR-values for one HDL-increasing allele;
Significant SNP associations after Bonferroni correction with eGFR are marked in grey
The p-value for SNPs showing a genome-wide significant association with eGFR are written in bold

Supplementary Table II: List of all 28 SNPs, which showed potential pleiotropic effects according to the GWAs catalogue (https://www.ebi.ac.uk/gwas/) [17]

SNP	Nearest Gene	Other phenotypes*
rs1121980	*FTO*	Triglycerides
rs11613352	*R3HDM2*	Triglycerides
rs12678919	*LPL*	Triglycerides
rs12748152	*PIGV*	Triglycerides
rs13107325	*SLC39A8*	BMI, blood pressure, hypertension
rs1532085	*LIPC*	Triglycerides
rs17145738	*TBL2*	Triglycerides
rs174546	*FADS1*	Triglycerides
rs1883025	*ABCA1*	Total cholesterol, metabolic syndrome
rs1936800	*RSPO3*	Triglycerides
rs2925979	*GAN*	Adiponectin, waist-hip-ratio
rs2954029	*TRIB1*	Triglycerides
rs2972146	*IRS1*	Triglycerides
rs3764261	*CETP*	Triglycerides
rs4420638	*APOC1*	LDL cholesterol, triglycerides, CRP, longevity
rs4765127	*ZNF664*	Triglycerides
rs4846914	*GALNT2*	Triglycerides
rs4917014	*IKZF1*	Lupus
rs581080	*TTC39B*	Total cholesterol
rs6065906	*PCIF1*	Triglycerides
rs6450176	*ARL15*	Adiponectin
rs7241918	*LIPG*	Total cholesterol
rs731839	*PEPD*	Triglycerides
rs7941030	*UBASH3B*	Total cholesterol
rs964184	*ZNF259*	Triglycerides
rs970548	*MARCH8*	Total cholesterol
rs998584	*VEGFA*	Triglycerides
rs9987289	*PPP1R3B*	Total cholesterol, LDL cholesterol

* Genome-wide associated with other phenotypes besides HDL cholesterol according to the GWAs catalogue

Supplementary Figure I: Schematic overview on the study design, main findings and interpretation of the results.

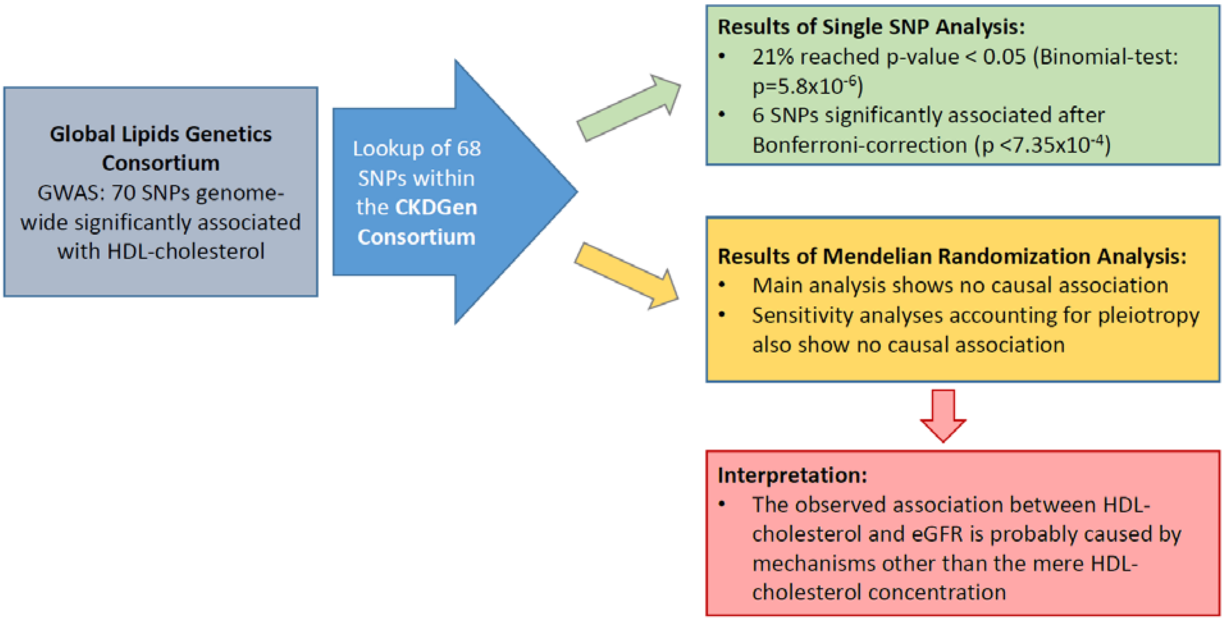

Supplementary Figure II: Funnel plot including the two-sample IVW-MR estimate (green) as well as the causal estimate obtained by MR-Egger regression (red) using all 68 SNPs that showed a genome-wide significant association with HDL-c and were available in both datasets used for the Mendelian randomization. The causal estimate is represented by the box and the vertical lines, and the corresponding 95%-CI is shown by the horizontal lines.

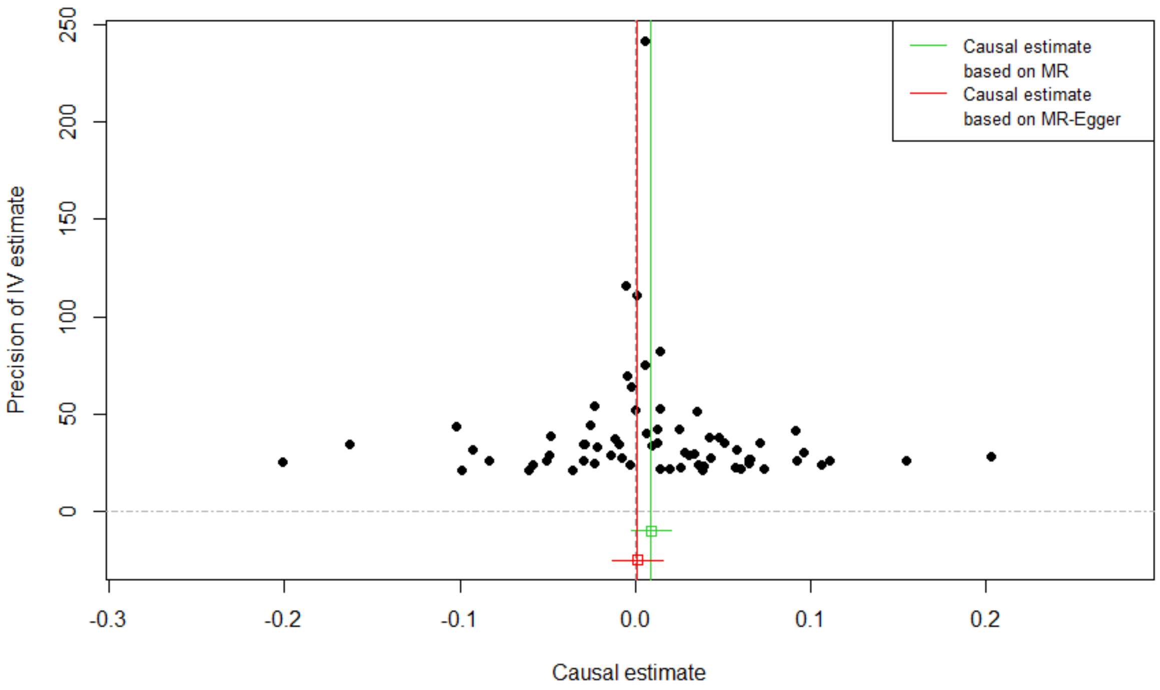

Supplementary Figure III: Scatterplots showing the effect estimates of SNP-HDL associations (+/- 95% CI) on the x- and SNP-eGFR associations (+/- 95% CI) on the y-axis. SNPs showing potential pleiotropic effects (according to the GWAS catalog) are marked in blue. The continuous black line represents the two-sample Mendelian Randomization estimate of HDL on eGFR, the dashed lines the corresponding 95% CI. For the Mendelian randomization estimate, potentially pleiotropic SNPs were excluded.

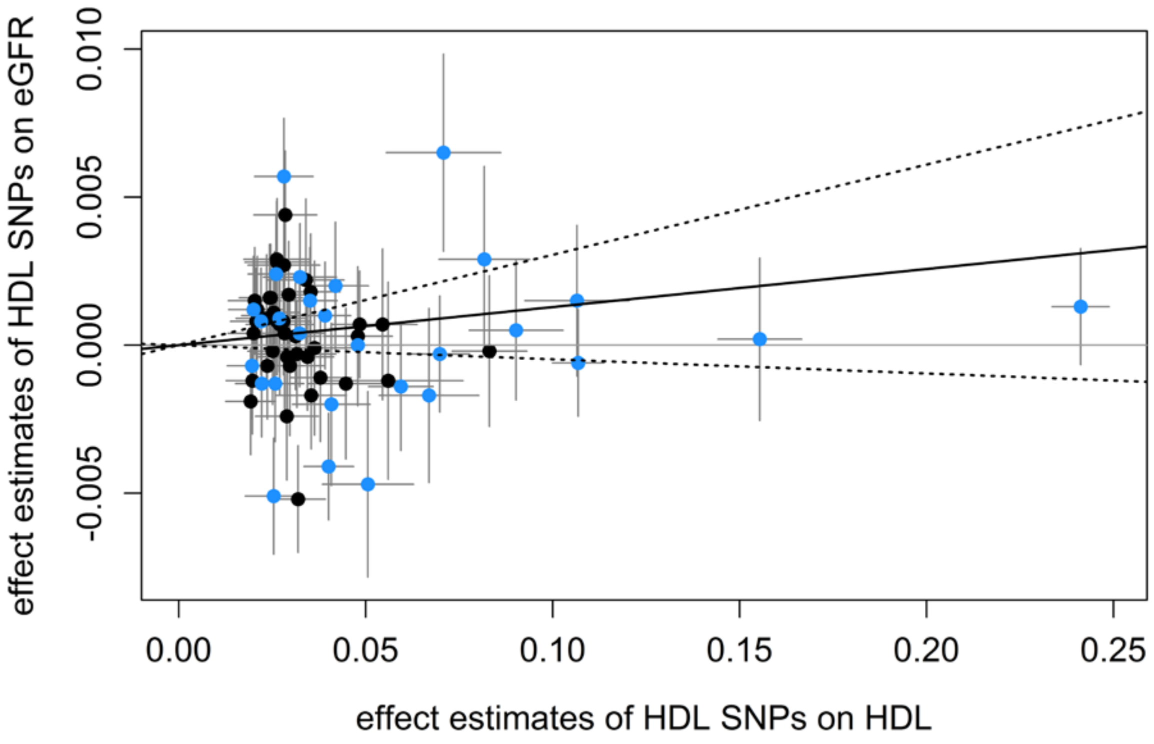

References

(1) Soubeyrand S, Martinuk A, Naing T, Lau P, McPherson R. Role of Tribbles Pseudokinase 1 (TRIB1) in human hepatocyte metabolism. Biochimica et Biophysica Acta 1862, 223-232. 2016.

(2) Bauer RC, Yenilmez BO, Rader DJ. Tribbles-1: a novel regulator of hepatic lipid metabolism in humans. Biochem.Soc.Trans. 43, 1079-1084. 2015.

(3) Iwamoto S, Boonvisut S, Makishima S, Ishizuka Y, Watanabe K, Nakayama K. The role of TRIB1 in lipid metabolism; from genetics to pathways. Biochem.Soc.Trans. 43, 1063-1068. 2015.

(4) Neuwirt H, Perco P, Kainz A, Muhlberger I, Leierer J, Braniff SJ, Mayer B, Mayer G, Rudnicki M. A 3-biomarker-panel predicts renal outcome in patients with proteinuric renal diseases. BMC.Med.Genomics 7, 75. 2014.

(5) Ashton-Chess J, Giral M, Mengel M et al. Tribbles-1 as a novel biomarker of chronic antibody-mediated rejection. Journal of the American Society of Nephrology 19, 1116-1127. 2008.

(6) Divers J, Palmer ND, Lu L, Langefeld CD, Rocco MV, Hicks PJ, Murea M, Ma L, Bowden DW, Freedman BI. Gene-gene interactions in APOL1-associated nephropathy. Nephrology, Dialysis, Transplantation 29, 587-594. 2014.

(7) Annema W, Dikkers A, Freark de BJ, Dullaart RP, Sanders JS, Bakker SJ, Tietge UJ. HDL Cholesterol Efflux Predicts Graft Failure in Renal Transplant Recipients. Journal of the American Society of Nephrology 27, 595-603. 2016.

(8) Vaziri ND. HDL abnormalities in nephrotic syndrome and chronic kidney disease. Nat.Rev.Nephrol. 12, 37-47. 2016.

(9) Soffientini U, Caridis AM, Dolan S, Graham A. Intracellular cholesterol transporters and modulation of hepatic lipid metabolism: Implications for diabetic dyslipidaemia and steatosis. Biochimica et Biophysica Acta 1842, 1372-1382. 2014.

(10) Chasman DI, Fuchsberger C, Pattaro C et al. Integration of Genome-Wide Association Studies with Biological Knowledge Identifies Six Novel Genes Related to Kidney Function. Hum.Mol.Genet. 21, 5329-5343. 8-9-2012.

(11) Pattaro C, Teumer A, Gorski M et al. Genetic associations at 53 loci highlight cell types and biological pathways relevant for kidney function. Nat.Commun. 7, 10023. 2016.

(12) Yang Q, Kottgen A, Dehghan A et al. Multiple genetic loci influence serum urate levels and their relationship with gout and cardiovascular disease risk factors. Circ.Cardiovasc.Genet. 3, 523-530. 2010.

(13) Köttgen A, Albrecht E, Teumer A et al. Genome-wide association analyses identify 18 new loci associated with serum urate concentrations. Nat.Genet. 45, 145-154. 2013.

(14) Richards JB, Waterworth D, O'Rahilly S et al. A genome-wide association study reveals variants in ARL15 that influence adiponectin levels. PLoS.Genet. 5, e1000768. 2009.

(15) Wilhelm LP, Tomasetto C, Alpy F. Touche! STARD3 and STARD3NL tether the ER to endosomes. Biochem.Soc.Trans. 44, 493-498. 15-4-2016.

(16) Willer CJ, Schmidt EM, Sengupta S et al. Discovery and refinement of loci associated with lipid levels. Nat.Genet. 45, 1274-1283. 2013.

(17) Welter D, MacArthur J, Morales J, Burdett T, Hall P, Junkins H, Klemm A, Flicek P, Manolio T, Hindorff L, Parkinson H. The NHGRI GWAS Catalog, a curated resource of SNP-trait associations. Nucleic Acids Res. 42, D1001-D1006. 2014.
