# Supplementary material for: Is High-Density Lipoprotein Cholesterol Causally Related to Kidney Function? Evidence From Genetic Epidemiological Studies
Source: Arterioscler Thromb Vasc Biol. 2016 Oct 26;36(11):2252–8. doi: 10.1161/ATVBAHA.116.308393 (PMC5084637; doi:10.1161/ATVBAHA.116.308393)
Supplement: Supplementary file 3 [file atv-36-2252-s003.pdf]

Methods

Data sources and SNP selection

To select genetic variants associated with HDL-cholesterol, published data from the Global Lipids Genetics Consortium (GLGC) [1] were used. The GLGC meta-analyzed data from 23 studies of European ancestry (n=94,595 individuals), which were genotyped with genome-wide SNP arrays and additionally 37 studies (n=93,982 individuals) which were genotyped with the Metabochip array [2]. For each locus that was found to be associated with HDL-cholesterol with SNPs having a p-value $<5x10^{-8}$, the lead SNP was selected, resulting in a list of 70 independent SNPs (sample size ranging from 92,820 to 187,141). We decided not to include SNPs that were found to be genome-wide significant exclusively with other lipid phenotypes than HDL cholesterol. With this approach weak instrument bias can be held as small as possible. For all SNPs, beta effect estimates, standard errors and genomic control corrected p-values were retrieved from the original publication. Beta effects refer to standard deviation of inverse-normally transformed HDL-cholesterol values for each increase in number of the effect allele. The effect allele is defined as the allele that increases HDL-cholesterol.

Publicly available GWAS summary statistics from the CKDGen Consortium were retrieved, and all 70 selected HDL-cholesterol SNPs were looked up for the association of these SNPs with kidney function parameters [3]. This consortium combined genome-wide data from up to 133,413 individuals of European ancestry from 49 predominantly population-based studies. The mean age of these studies ranged from 37 to 81 years and all studies combined included on average 54.8% women. The estimated glomerular filtration rate (eGFR) based on a creatinine measurement was estimated using the four-variable Modification of Diet in Renal Disease Study Equation and the mean values for the studies ranged from 71.2 to 104.8 ml/min/1.73m^2. Chronic kidney disease (CKD) was defined by an eGFR <60 ml/min/1.73m^2 and was present in 12,385 individuals. The study sample included 11,522 cases with diabetes and 54,824 cases with hypertension. A sample characteristic and a short description for each study cohort were provided in the Supplementary Tables 1 and 16 of Pattaro et al. [3].

Summarized meta-analysis results (beta estimates, for genomic control corrected standard errors and genomic control corrected p-values) were available for eGFR based on serum creatinine (eGFR). 68 of the 70 selected SNPs were available, with a sample size ranging from 122,575 to 133,808. Beta effects refer to change in log-transformed eGFR for each increase in number of the effect allele. The direction of effect estimates were aligned to the HDL-increasing allele. In both consortia, meta-analysis was performed using inverse

variance-weighted fixed effect models. Between the two consortia, there is a partial overlap: 15 studies contributed to both, the GLGC and CKDGen Consortia, including data from about 58,000 participants.

Statistical Methods

For all individual SNPs, p-values from the association on log-transformed eGFR values are presented. Overrepresentation of p-values smaller than 0.05 was tested using a binomial test. The significance level of the single SNP look-up was set to $0.05/68=7.35\times10^{-4}$ after Bonferroni correction on the number of SNPs.

The Mendelian randomization analysis was performed using the published summarized data as described in [4]. The independence of SNPs was assessed using SNiPA (http://snipa.helmholtz-muenchen.de/snipa/) [5]. The ratio estimates from all 68 SNPs were combined using the fixed-effects meta-analysis model with inverse variance weights as proposed by Burgess et al. [4]. The inverse variance weighted ratio estimate can also be interpreted as a weighted regression from the HDL-cholesterol effect estimates of the HDL-cholesterol SNPs on the eGFR effect estimates of the same SNPs (removing the intercept). A random-effects model was applied when significant heterogeneity was detected based on the Cochran's Q statistic [6]. Since we observed a substantial overlap between the two consortia of about 43% we included the random effects model corrected for the overlap as well as the standard random effects model approach as recommended by Burgess et al. (only available in online software code:
http://www.mendelianrandomization.com/index.php/software-code).
To assess the strength of the instrumental variables (SNPs), the proportion of the phenotypic variance of HDL-cholesterol explained by the HDL-cholesterol SNPs was estimated as given in Pattaro et al. [3;7]. Using their formula, the percentage of phenotypic variance explained can be estimated as $\sum_{i=1}^{68} R_i^2$, where $R_i^2 = \beta_i^2 var(SNP_i)/var(HDL)$ is the coefficient of determination for all SNPs associated with HDL-cholesterol, β_i is the estimated effect of the ith SNP on HDL in s.d., $var(SNP_i) = 2 \times MAF_{SNP_i} \times (1 - MAF_{SNP_i})$ and $var(HDL) = 1$, since the beta estimates refer to change in 1 s.d. of HDL-cholesterol.

A central assumption of the Mendelian randomization approach is that SNPs used as instrumental variables should not have pleiotropic effects. Therefore, different methods were used to detect possible pleiotropy and also to account for it:

1) The MR-Egger regression to assess directional pleiotropy
2) The Weighted median estimation method
3) The Exclusion of possible pleiotropic SNPs as sensitivity analysis
   a) based on the gtx package in R

b) based on the GWAS catalog

4) Adjusting for the effect estimates of HDL-cholesterol SNPs on LDL-cholesterol and triglycerides

MR-Egger regression [8] was used to investigate whether there is directional bias caused by pleiotropy. Directional bias means that the pleiotropic effects of genetic variants are not balanced about the null and are drawn into one direction. This regression is an adaption of the standard Egger regression which is used to analyze small study bias in the meta-analysis literature. The intercept obtained from the MR-Egger regression gives an estimate of directional bias and the slope coefficient provides an estimate of the causal effect, which is consistent even when all the genetic variants are invalid instrumental variables with respect to pleiotropy [8]. Additionally, the weighted median estimator was calculated as proposed by Bowden et al. [9]. In this method, the ratio estimates are ordered and weighted by the inverse of their variance. The weighted median estimator is then the median of these estimates, according to the weights. This estimator is consistent if at least 50% of the weight comes from valid instrumental variables (IV). Although the MR-Egger regression method allows all the IVs to be invalid, the weighted median approach offers the advantages of an improved precision compared to the MR-Egger regression. Therefore, both methods were used to assess whether pleiotropy had influenced our results.

In a further sensitivity analysis, all SNPs that were assumed to have pleiotropic effects were excluded. Each SNP was screened for association with other phenotypes in the NHGRI-EBI GWAS Catalog (https://www.ebi.ac.uk/gwas/) [10] and a sensitivity analysis was performed excluding such SNPs, which were associated with other phenotypes than HDL-cholesterol. However, bias in Mendelian randomization only occurs due to pleiotropy, when the SNPs are associated with other phenotypes, which also influence the outcome variable (in our case: eGFR) or are independently associated with the outcome variable itself. If this is not the case and there is also no direct effect of the SNPs with the outcome variable, the effect of the SNPs on the outcome is mediated completely by the intermediate variable (in our case: HDL-cholesterol concentrations). Then, the causal effects of all SNPs individually should rather be homogeneous and approximate the true unknown causal effect of HDL-cholesterol on eGFR [6;11;12]. This assumption was tested by a goodness of fit test using the function “grs.filter.Qrs” in package “gtx” in R (Johnson, T.: Efficient Calculation for Multi-SNP Genetic Risk Scores. Poster presentation at the American Society of Human Genetics Annual Meeting, San Francisco, 2012). This function performs a stepwise downward “model selection” in which SNPs are iteratively removed from the risk score until the heterogeneity test is no longer significant at the specified threshold ($p_{threshold}$=0.05). An illustration of this approach is given in Figure 4. SNPs showing a deviation from this assumption and are

therefore potentially not mediated completely by HDL-cholesterol (as SNP 5 in Figure 4) were excluded in a further sensitivity analysis.

As it might be too strict to exclude the SNPs that seem to have pleiotropic effects, we also included a linear regression adjusted for LDL-cholesterol and triglycerides as in Do et al. [13]. For this analysis, effect estimates for all selected HDL-C SNPs on Triglycerides and LDL-C were derived from Willer et al. [14]. Using this approach, it is possible to adjust for the effects the HDL-cholesterol SNPs might have on LDL-cholesterol and triglycerides.

References

1. Willer CJ, Schmidt EM, Sengupta S et al. Discovery and refinement of loci associated with lipid levels. *Nat.Genet.* 2013; 45:1274-1283.
2. Voight BF, Kang HM, Ding J et al. The metabochip, a custom genotyping array for genetic studies of metabolic, cardiovascular, and anthropometric traits. *PLoS.Genet.* 2012; 8:e1002793.
3. Pattaro C, Teumer A, Gorski M et al. Genetic associations at 53 loci highlight cell types and biological pathways relevant for kidney function. *Nat.Commun.* 2016; 7:10023.
4. Burgess S, Butterworth A, Thompson SG. Mendelian randomization analysis with multiple genetic variants using summarized data. *Genet.Epidemiol.* 2013; 37:658-665.
5. Arnold M, Raffler J, Pfeufer A et al. SNiPA: an interactive, genetic variant-centered annotation browser. *Bioinformatics.* 2015; 31:1334-1336.
6. Greco MF, Minelli C, Sheehan NA et al. Detecting pleiotropy in Mendelian randomisation studies with summary data and a continuous outcome. *Stat.Med.* 2015; 34:2926-2940.
7. Rosner B. Fundamentals in biostatistics. Thomson-Brooks/Cole; Belmont, CA; 2006.
8. Bowden J, Davey SG, Burgess S. Mendelian randomization with invalid instruments: effect estimation and bias detection through Egger regression. *Int.J.Epidemiol.* 2015; 44:512-525.
9. Bowden J, Davey SG, Haycock PC et al. Consistent Estimation in Mendelian Randomization with Some Invalid Instruments Using a Weighted Median Estimator. *Genet.Epidemiol.* 2016; 40:304-314.
10. Welter D, MacArthur J, Morales J et al. The NHGRI GWAS Catalog, a curated resource of SNP-trait associations. *Nucleic Acids Res.* 2014; 42:D1001-D1006.
11. Zhang C, Doherty JA, Burgess S et al. Genetic determinants of telomere length and risk of common cancers: a Mendelian randomization study. *Hum.Mol.Genet.* 2015; 24:5356-5366.
12. Burgess S, Scott RA, Timpson NJ et al. Using published data in Mendelian randomization: a blueprint for efficient identification of causal risk factors. *Eur.J.Epidemiol.* 2015; 30:543-552.
13. Do R, Willer CJ, Schmidt EM et al. Common variants associated with plasma triglycerides and risk for coronary artery disease. *Nat.Genet.* 2013; 45:1345-1352.
14. Willer CJ, Schmidt EM, Sengupta S et al. Discovery and refinement of loci associated with lipid levels. *Nat.Genet.* 2013; 45:1274-1283.
